# Advancing Intersectional Discrimination Measures for Health Disparities Research: Protocol for a Bilingual Mixed Methods Measurement Study

**DOI:** 10.2196/30987

**Published:** 2021-08-30

**Authors:** Ayden I Scheim, Greta R Bauer, João L Bastos, Tonia Poteat

**Affiliations:** 1 Department of Epidemiology and Biostatistics Dornsife School of Public Health Drexel University Philadelphia, PA United States; 2 Department of Epidemiology and Biostatistics Schulich School of Medicine and Dentistry Western University London, ON Canada; 3 Department of Public Health Federal University of Santa Catarina Florianópolis Brazil; 4 Department of Social Medicine University of North Carolina at Chapel Hill Chapel Hill, NC United States

**Keywords:** stigma, discrimination, racism, measurement, health disparities, survey research, psychometrics

## Abstract

**Background:**

Guided by intersectionality frameworks, researchers have documented health disparities at the intersection of multiple axes of social status and position, particularly race and ethnicity, gender, and sexual orientation. To advance from identifying to intervening in such intersectional health disparities, studies that examine the underlying mechanisms are required. Although much research demonstrates the negative health impacts of perceived discrimination along single axes, quantitative approaches to assessing the role of discrimination in generating intersectional health disparities remain in their infancy. Members of our team recently introduced the Intersectional Discrimination Index (InDI) to address this gap. The InDI comprises three measures of enacted (day-to-day and major) and anticipated discrimination. These attribution-free measures ask about experiences of mistreatment *because of who you are*. These measures show promise for intersectional health disparities research but require further validation across intersectional groups and languages. In addition, the proposal to remove attributions is controversial, and no direct comparison has ever been conducted.

**Objective:**

This study aims to cognitively and psychometrically evaluate the InDI in English and Spanish and determine whether attributions should be included.

**Methods:**

The study will draw on a preliminary validation data set and three original sequentially collected sources of data: qualitative cognitive interviews in English and Spanish with a sample purposively recruited across intersecting social status and position (gender, sexual orientation, race and ethnicity, socioeconomic status, age, and nativity); a Spanish quantitative survey (n=500; 250/500, 50% sexual and gender minorities); and an English quantitative survey (n=3000), with quota sampling by race and ethnicity (Black, Latino/a/x, and White), sexual or gender minority status, and gender.

**Results:**

The study was funded by the National Institute on Minority Health and Health Disparities in May 2021, and data collection began in July 2021.

**Conclusions:**

The key deliverables of the study will be bilingual measures of anticipated, day-to-day, and major discrimination validated for multiple health disparity populations using qualitative, quantitative, and mixed methods.

**International Registered Report Identifier (IRRID):**

PRR1-10.2196/30987

## Introduction

### Health Disparities

Disparities in health status and access to care along single axes of social status and position (SSP), such as race and ethnicity, gender, and sexual orientation, are well documented in the United States [1,2]. Informed by intersectionality frameworks, recent research has emphasized disparities at the intersection of multiple axes of SSP. Intersectionality is rooted in Black feminist scholarship [3-5] and has become a central framework for public health efforts to understand and intervene in multiple, interacting, and context-dependent forms of social and health advantage and disadvantage [6,7]. Intersectional health disparities research allows for independent estimation of outcomes across intersections, acknowledging that groups at particular SSP intersections may experience better or poorer health than that predicted by nonintersectionally combining the effects of individual SSP. Examples include HIV infection among Black sexual minority men and transgender women [8], smoking among Asian or Pacific Islander sexual minority women [9], opioid misuse among high-income Black women [10], and hypertension among Black and Latina women [11].

### Health Impacts of Perceived Discrimination

A robust literature demonstrates that perceived, self-reported discrimination is associated with poorer mental and physical health, with most studies focusing on the health impacts of racial and ethnic discrimination among people of color and, to a lesser extent, sexuality- and gender-based discrimination among lesbian, gay, bisexual, and transgender people [12-15]. Perceived discrimination, including acute *major* events, chronic *day-to-day* discrimination, and anticipated discrimination, are thought to primarily impact health through (1) stress processes resulting in distress or health-harming coping strategies, and (2) physiological reactions such as elevated blood pressure and dysregulated hypothalamic-pituitary-adrenal axis function, which elevate cardiovascular disease risks [16-20]. Although not amenable to self-reporting and thus beyond the scope of this project, structural forms of oppression, such as residential segregation and lack of legal protection, set the context for perceived discrimination and drive poor health [21,22].

### Need for Intersectional Discrimination Measures

Quantitative intersectionality studies have burgeoned in the last decade and have largely focused on describing inequalities across groups cross stratified by SSP, taking an intercategorical [6] approach by examining outcomes across multiple intersecting SSPs [23,24]. In contrast, intersectional studies of the mechanisms potentially underlying health disparities have largely been intracategorical studies, focusing on experiences at a particular intersection [25,26]. Recognizing the conceptual bias inherent in using within-group analyses to make inferences about between-group differences [27,28], we and others have called for analytic approaches to *intercategorical* intersectionality to identify modifiable processes, including discrimination, that lead to health disparities [27-30]. Such studies require discrimination measures whose estimates are meaningfully comparable across intersectional groups. Previous intercategorical studies have adopted measures of racial and ethnic discrimination [31-36] that may not have content validity for other types of discrimination, or even for diverse racial and ethnic groups [37,38]. In fact, recent evidence shows that traditional scales of discrimination may not provide meaningful estimates of perceived general or racial and ethnic discrimination across diverse groups in the United States [39].

### Intersectional Discrimination Index

The Intersectional Discrimination Index (InDI) is a set of three measures of anticipated (InDI-A) and enacted (day-to-day [InDI-D] and major [InDI-M]) discrimination, originally designed for cross-group use. Notably, although anticipated discrimination is associated with mental health and cardiovascular stress responses [40,41], it is rarely measured [42]; the InDI-A is the first scale to measure this construct across multiple SSP. The InDI asks about discrimination *because of who you are* but without requesting attributions to specific SSP. Rather, these measures can be analyzed using cross-stratified demographic data. In 2019, our team published a binational study that provided preliminary evidence of the measures’ construct validity and test-retest reliability. Among participants in the United States (n=1518) and Canada (n=1065), as hypothesized, people of color and sexual and gender minorities (SGMs) reported higher levels of perceived discrimination, lifetime and past-year discrimination were associated with psychological distress, and enacted discrimination was correlated with the Everyday and Major Discrimination scales [43,44]. Test-retest reliability at 6 weeks was 0.70-0.72, similar to or higher than comparable measures [45,46].

The preliminary InDI study was published alongside four invited commentaries that identified the strengths of the InDI as well as areas requiring further study to clarify its utility for intersectional health disparities research [25,47-49]. Qualitative research using cognitive interviewing would reveal how respondents understand and respond to items in relation to multiple SSPs, identifying items that may require revision [48]. Psychometric analyses are needed to further validate the InDI measures across intersectional groups, including the assessment of configural, metric, and scalar properties (eg, factor structure, item loadings, and item intercepts) and measurement equivalence [26,47]. The preliminary InDI study was ethnically and racially diverse but had small sample sizes at specific intersections (eg, 50 Black SGM in the combined US and Canada sample), precluding intersectional validation analyses. In addition, Spanish measures are essential for health disparities research in the United States but need to be assessed for conceptual, semantic, and measurement equivalence to allow valid data pooling and comparison [50,51]. This is critically important given evidence of systematic response differences to discrimination measures by survey language and acculturation among Latino/a/x persons [52,53].

### Asking for Attribution in Intersectional Discrimination Measures

To date, intercategorical, intersectional discrimination studies have adapted extant measures to permit multiple attributions [31-36]. Research has highlighted attributional ambiguity among individuals situated at the intersection of marginalized SSP [54-56]. Allowing multiple attributions may not reduce cognitive burden or improve measurement validity; for example, conceptual slippage between SSPs may make participant selections arbitrary [56]. Options for quantitatively modeling attributions are limited because they are not tied to frequencies; this has resulted in analyses that either dichotomize discrimination or count attributions [31-36,57], representing losses of information on dose-response relationships [58] and on the complexity of experiences at particular intersections [26]. Therefore, the InDI was designed to be attribution free. This decision rests on a lack of evidence that the health impacts of discrimination depend on attribution. Some researchers have argued that a different construct underlies measures of discrimination that include attributions to perceived discrimination compared with those that do not [59], but no study has empirically tested the validity of this claim. Indeed, a 2015 review noted that the few studies comparing attributed and unattributed (racial and ethnic) discrimination measures have compared *apples and oranges* (ie, measures that reflect different constructs); the authors called for direct comparisons, including cognitive interviewing [18]. Further, some research participants reported the ability to unambiguously attribute discrimination to one or more SSP, and health disparities researchers remain interested in the burden and consequences of specific discrimination types [47,56]. Thus, we will conduct an evaluation inclusive of participant perspectives, examination of the degree to which SSP and attributions overlap, and direct comparison of correlations with health outcomes.

### Objectives

The primary aims of the study are to (1) assess the content and wording of the InDI measures (InDI-A, InDI-D, InDI-M) in English and Spanish; (2) evaluate the InDI measures’ configural, metric, and scalar structures, as well as their measurement equivalence across language and intersecting SSP; and (3) determine whether attributions should be included in the InDI measures.

## Methods

### Overview of Study Design

As described in Table 1, the study will draw on the preliminary validation data set and three original data sources.

**Table 1:** Overview of approach.

Method	Sample	Aims	Analysis
Cognitive interviews (n=50)	25 (50% per language) sampled for maximum diversity	1: Cognitive evaluation and InDI^a^ revisions 2: Participant perspectives on attributions	1: Within- and between-interview analysis using Q-Notes 2: Qualitative analysis using grounded theory techniques
Previously collected validation data set (n=2583)	1518 (58.77%) in the United States 1065 (41.23%) in Canada	2: Intersectional psychometric evaluation	2: Exploratory factor analysis, exploratory structural equation modeling, and CFA^b^
Quantitative surveys	500 in Spanish^c^ 3000 in English^c^	2: Intersectional psychometric evaluation 3: Determine analytic utility of attributions	2: Multiple indicator multiple cause models and multigroup CFA 3: Descriptive statistics and multivariable regression models

^a^InDI: Intersectional Discrimination Index.

^b^CFA: confirmatory factor analysis.

^c^Quota sampling by race and ethnicity and sexual or gender minority status.

### Participants

Eligible participants will be aged ≥18 years and residing in the United States. For cognitive interviews (25/50, 50% English; 25/50, 50% Spanish), participants will be of any race and ethnicity and purposively sampled to achieve maximum diversity in gender, sexual orientation, race and ethnicity, socioeconomic status, age, and nativity. Quota sampling will be used for the quantitative surveys. The Spanish survey (n=500) will include 250 SGM and 250 non-SGM. The English survey (n=3000) will include non-Hispanic Black (1000/3000, 33%), non-Hispanic White (1000/3000, 33%), and Hispanic or Latino/a/x persons of any race (1000/3000, 33%), with 50% (250/500 for Spanish and 1500/3000 for English) of each group being composed of SGM. We will further stratify recruitment by gender to generate sample sizes of approximately 250 at each race and ethnicity *SGM* gender intersection (eg, White non-SGM women). Transgender and gender nonbinary respondents will be grouped by gender identity (eg, transgender men and transmasculine persons with men).

### Recruitment

Participants will be recruited using Facebook and Google advertisements in English and Spanish. Advertisements will also be placed on Black-, Latino/a/x-, and SGM-focused websites and circulated through relevant organizations. For the cognitive interviews, ads will link to the study information website. Interested individuals will complete a demographic screener and provide contact information. The bilingual study staff will contact selected participants to schedule an interview. For the quantitative survey, the ad will link to an eligibility screener with programmed quotas; individuals who meet eligibility criteria and whose recruitment category is open will be invited to participate and directed to the consent page. Multiple evidence-based strategies will be used to prevent and detect fraudulent respondents, including nondisclosure of eligibility criteria in ads, modest incentives, blocking responses from the same IP address, CAPTCHA (Completely Automated Public Turing test to tell Computers and Humans Apart), consistency and attention checks, and exclusion of records with implausible response times [60-62]. Using Qualtrics (SAP Inc) software, the English and Spanish surveys will be programmed as a single bilingual survey to prevent duplicate participation. Cognitive interview and survey participants will receive US $50 and US $15 electronic Amazon gift cards, respectively.

### Cognitive Interviews

Interviews will be conducted by trained research assistants using Health Insurance Portability and Accountability Act—compliant videoconferencing software (Zoom Health Insurance Portability and Accountability Act Private Mode). Verbal probing will be used to assess potential problems in the cognitive processes of question comprehension, information retrieval, judgment and estimation, and response [63]. At the start of the interview, participants will be sent a weblink to a questionnaire containing the unattributed InDI. Participants will respond to each item, and then the interviewer will ask them how they answered the question and why. Following best practices for cognitive interviewing, subsequent probes will ask the respondent to paraphrase the question, judge their confidence in the response, reflect on the accuracy of their recall, and indicate whether they had difficulty answering [64].

After completing the unattributed measures, participants will be presented with one of two attributed versions, one that requests either item-level attributions or overall attributions for each of the InDI measures (Multimedia Appendix 1). The interviewer will ask the respondent to complete the attribution items and then probe their responses as above. In addition, participants will be asked to share their perspectives on the importance of attributions (eg, “Did you prefer answering these questions with or without being asked for reasons why others mistreated you?”).

Interviewers will take notes directly into Q-Notes software, developed by the National Center for Health Statistics for the analysis of cognitive interviewing data [65]. Q-Notes is designed to facilitate real-time cross-site collaborations to evaluate instruments in multiple languages. This will facilitate the rapid analysis of data for aim 1 and subsequent revisions to improve the clarity and completeness of the InDI items. Interviews will also be recorded, transcribed verbatim, and translated into English if needed.

### Quantitative Surveys

The self-administered survey will take approximately 20 minutes to complete.

#### Intersectional Discrimination Index

All Spanish-language participants will receive the unattributed InDI (Multimedia Appendix 1), with potential modifications based on cognitive interview findings. For the English survey, we will use the block-randomization feature in Qualtrics to implement a split-ballot design: 1000 participants will receive each of the unattributed, item-level attributed, or overall attributed versions of the InDI.

#### Demographics

Age, sex assigned at birth, current gender identity, race, ethnicity, immigration history, education, income, sexual orientation, geographic region, and community size (eg, urban and rural) data will be collected.

#### Health Outcomes

To permit psychometric evaluation of these novel instruments (aim 2) and comparisons between attribution methods (aim 3), analyses in the current proposal will focus on two of the most well-established health consequences of perceived discrimination: psychological distress and self-rated general health [15]. Psychological distress will be measured using the 6-item K6 measure developed by Kessler et al [66]. The K6 was developed to estimate the prevalence of serious mental illness and has shown good sensitivity and excellent specificity in US population samples when dichotomized at a score of 13/24 or above and compared with Diagnostic and Statistical Manual of Mental Disorders-IV diagnoses via structured clinical interviews [66,67]. Self-rated general health, a robust predictor of morbidity and mortality [68], will be assessed using a standard question from the Behavioral Risk Factor Surveillance System and other federal surveys (“Would you say in general that your health is—excellent, very good, good, fair, or poor?”). To facilitate secondary analyses of these data focused on a wider range of health outcomes, the survey will also collect data on substance use (nicotine, alcohol, and illicit drugs), hypertension, and diabetes.

### Analysis Plan

#### Aim 1: Cognitive Evaluation of the InDI Measures in English and Spanish

Using sorting features in the Q-Notes software, we will first analyze detailed interview notes at the within-interview level (to capture response errors). Next, we will compare data on each survey item across interviews to evaluate consistency in understanding what the question intends to capture. Finally, we will sort data by participant subgroups (eg, by language, race and ethnicity, and SGM status individually) to identify potential response biases. Two analysts will complete each of these steps independently, meeting to compare analysis notes and generate a list of potential modifications at the end of each stage. Potential modifications to address identified problems with instructions, items, or response options will be reviewed by all team members; if modifications are made, the revised measures will be evaluated with five new cognitive interviews in each language.

#### Aim 2: Psychometric Evaluation of the InDI Measures Across Languages and Intersecting SSPs

Analyses will be conducted in MPlus (Muthén & Muthén) and Stata (StataCorp LLC). As a first step, we will conduct exploratory factor analysis, exploratory structural equation modeling, and confirmatory factor analysis (CFA) of the InDI-D and InDI-M in our previously collected data, using split-halves of the sample by country (n=1065 and n=1518). These analyses will help us establish the configural and metric structures of the two enacted discrimination measures. We previously used this approach to assess the psychometric properties of the InDI-A, finding support for its hypothesized unidimensionality. At that time, we opted not to conduct factor analyses of the enacted discrimination measures, with the rationale that their items are causal-formative indicators that aggregate to form a construct, rather than items that reflect the level of an underlying construct and should necessarily correlate [69]. Nevertheless, existing research demonstrates that discrimination scales do show strong interitem correlations and factor structures [47], likely reflecting both respondent characteristics that determine perception and self-reporting of discrimination, as well as the social clustering of discrimination. Furthermore, the assessment of configural, metric, and scalar structures of the InDI measures should precede the evaluation of measurement equivalence across intersectional groups, thereby determining whether the discrimination burdens they estimate can be meaningfully compared across groups [47]. We will evaluate the goodness of fit for CFA with parsimony, incremental, and absolute indices [70]. If substantive changes are made to the InDI items in aim 1, we will instead conduct the exploratory factor analysis, exploratory/confirmatory factor analysis, and CFA using randomly selected samples of the new survey data (n=200 each).

Once the configural and metric structures of the InDI measures are established, we will assess their scalar structures. We will estimate the Loevinger H coefficient to determine whether scales reflect the Mokken model (ie, those endorsing severe items are more likely to endorse less severe items) [71]. This will be followed by the estimation of item response theory models [72]; item discrimination and item difficulty will be estimated to assess how well the InDI measures tap their underlying traits. The results will be graphically displayed in a Wright map, item characteristic curves, a test information function plot, and a test characteristic curve graph.

Next, we will use originally collected survey data to assess differential item functioning and measurement equivalence, using Multiple Indicator Multiple Cause (MIMIC) models and multigroup CFA (mCFA), respectively. These approaches are complementary: mCFA compares the full measurement model across groups, whereas MIMIC is generally limited to testing for differences in item difficulty (thresholds for endorsement). However, mCFA estimates parameters separately for groups defined by a single categorical variable and requires a sufficient sample size for each group, whereas MIMIC uses a single model for the full sample, making it more efficient and permitting isolation of reasons for differential item functioning [73,74]. Therefore, we will use the MIMIC methods across a range of SSPs of interest and the mCFA methods across the intersectional quota-sampled strata. We will include gender, sexual orientation, race and ethnicity, socioeconomic status, age, and nativity as covariates in the MIMIC model for each measure.

We will use mCFA to determine whether the configural, metric, and scalar structures of the InDI measures are equivalent across (1) language (English vs Spanish) and (2) the 12 intersectional race and ethnicity*SGM*gender sample strata. Achieving equivalence across all these scale structures is necessary for the InDI measures to generate meaningful cross-group comparisons. After estimating the baseline models for each group, we will test configural equivalence across language and intersectional strata. We will then assess the metric equivalence of the scales by examining whether factor loadings vary across these groups by comparing the fit of the metric models with those of the configural models. To assess scalar equivalence, item thresholds will be tested for their equivalence among the same groups. Formal comparisons will be carried out by comparing the fit of the scalar models with those of the metric models. To compare less (eg, configural) and more restrictive (eg, metric and scalar) models, we will use a reduction ≥0.002 in the value of the comparative fit index as an unbiased indication of lack of equivalence, given the sensitivity of *P* values to large sample sizes [75]. We will also assess the partial invariance of the InDI measures. If needed (eg, because of numerous large modification indices), the alignment method will be used as an alternative to assess measurement equivalence [76].

#### Aim 3: Determine Whether Attributions Should Be Included in the InDI Measures

##### Overview

Aim 3 will use a convergent mixed methods design; quantitative and qualitative data analyses will be conducted separately and then the findings will be integrated to identify areas of convergence and divergence [77], following the National Institutes of Health Best Practices guidelines for mixed methods research [78].

##### Qualitative Analysis

Transcripts will be uploaded to Dedoose cloud-based software for analysis, facilitating remote collaboration. We will code the portion of the interview in which participants reflect on attribution items as well as any other portions relevant to attribution (eg, if raised by a participant earlier in the interview). The analysis will use techniques adapted from grounded theory [79,80]. Inductive coding to identify emergent themes will follow the constant comparative method of going back and forth between the data and coding to identify patterns and regularities. Two analysts will read all transcripts. Each analyst will code 5 transcripts independently, at which point discrepancies will be resolved through discussion before the remaining transcripts are coded. We will compare codes and themes by age, race, ethnicity, nativity, urban or rural residence, gender, and sexual orientation. To establish credibility, we will maintain an audit trail of coding decisions.

##### Quantitative Analysis

As a first step, we will compare discrimination burden (mean or median score on each of the InDI-A, InDI-D, and InDI-M) across the three conditions for each measure (unattributed, item-level attribution, and overall attribution) to determine whether the presence of attributions influences reporting. We will use appropriate parametric and nonparametric statistics (eg, chi-square and Mann-Whitney U test) to test for statistically significant differences between conditions. Next, we will estimate concordance between intersectional SSPs and discrimination attributions to determine the extent to which attributions align with SSPs. Among those reporting any discrimination in both the item-level and overall attribution conditions, we will calculate the proportion of (1) Black or Latino/a/x individuals who report racial and/or ethnic discrimination, (2) SGM individuals who report sexuality and/or gender identity–related discrimination, and (3) Black or Latino/a/x SGM who report both types of discrimination. In the item-level attribution condition, we will further calculate the proportion of items for which the aforementioned groups endorsed the respective attributions.

Finally, we will compare the magnitude of effect on psychological distress (continuous) and fair or poor (vs good or excellent) self-rated general health of reporting each discrimination type (InDI-A, InDI-D, and InDI-M), (1) based on sexuality and/or gender identity, (2) based on race and/or ethnicity, or (3) based on both (1) and (2). Using data from the attributed conditions, we will fit linear regression models to separately estimate the association between a categorical indicator of discrimination attributions (race and/or ethnicity, sexuality and/or gender, both, or other attributions only) and psychological distress, adjusting for total discrimination burden. These analyses will be conducted separately among Black and Latino/a/x SGM reporting any discrimination for each measure (InDI-A, InDI-D, and InDI-M). Parallel analyses focused on attributions to race and/or ethnicity, gender, both, or other attributions will be conducted among Black and Latino/a/x women. For self-rated health, we will use a similar approach with logistic regression.

##### Mixed Method Integration

We will merge qualitative and quantitative databases for analysis and interpretation. Specifically, we will use a joint display of data to visually represent and compare findings from each data set as they relate to key concepts [77,78,81]. We will be attentive to differences in the ethnoracial composition of the data sets to identify any themes that are unique to non-Black, non-Latino/a/x people of color. Implications for the measurement of divergent or inconsistent findings will be identified, with particular attention to how optimal measurement strategies might differ across intersectional groups and research foci.

### Sample Size

For cognitive interviews, our sample size exceeds qualitative research guidelines for achieving data saturation—the number of interviews by which no new relevant themes are identified (eg, up to 25 participants) [82,83]. The quantitative survey sample sizes were determined based on sample size requirements for multigroup CFA, which has the greatest sample size requirement among the planned analyses. Simulation studies show that 200 respondents per group is the minimum number required to have ≥80% power to detect measurement invariance via changes of ≥0.002 in the comparative fit index [75]. Therefore, we will recruit 250 per race and ethnicity*SGM*gender stratum in English (n=3000); the Spanish-language sample (n=500) will allow for mCFA by language as well as by SGM status, race, or gender. All other planned quantitative analyses have smaller sample size requirements.

## Results

The study was funded by the National Institute on Minority Health and Health Disparities from May 6, 2021, to January 31, 2023 (Multimedia Appendix 2). The study was approved by the institutional review board of Drexel University in May 2021 (IRB # 2006007889). Cognitive interview data collection began in July 2021. The publication of study results is expected to begin in early 2023.

## Discussion

### Study Importance

This study offers multiple innovations. It aligns with the National Institute on Minority Health and Health Disparities priorities for promoting the advancement of health disparities science by “strengthen[ing] the understanding of how racism and discrimination are conceptualized and measured, and how they contribute to health disparities” [84]. The key deliverables of this study will be bilingual measures of anticipated, day-to-day, and major discrimination validated for multiple health disparity populations using rigorous qualitative, quantitative, and mixed methods. Despite the high cognitive burden posed by discrimination measures, few studies have combined cognitive interviewing and psychometric approaches to validate them [85]. By subjecting these novel measures to a comprehensive and early evaluation of measurement equivalence, we will identify and be able to revise potentially problematic items before widespread use. Multiple studies have uncovered differential item functioning across diverse social groups among measures, such as the widely used Everyday Discrimination Scale [39,85,86], but only after they had been used in hundreds of studies [87] and were thus unlikely to be modified. The study will further advance measurement methods for health disparities research by determining the utility of including attributions in intersectional discrimination measures. If an attribution-free approach is viable, it will be possible to briefly assess discrimination experiences across a range of individual SSPs and their intersections in broad population surveys, enabling health disparities researchers to answer a much wider range of questions than with single-attribution measures.

### Limitations, Challenges, and Future Directions

Our 2-year study timeline is feasible because we have developed a two-stage analysis plan for cognitive interview data to ensure that any InDI revisions can be made rapidly, facilitating a timely launch of the quantitative survey. Our web-based recruitment and data collection plan will not be affected by social distancing requirements related to the COVID-19 pandemic. Nonrandom sampling will limit generalizability, but quota sampling will allow for the evaluation of InDI measures in intersectional strata that are typically small in representative surveys. The quantitative survey focuses on the largest racial and ethnic groups in the United States in which most health research on discrimination is conducted; as the design requires 1000 participants per racial or ethnic group, this restriction is necessary given finite resources. However, cognitive interviews will include all ethnoracial groups to ensure that revisions are made inclusively, and the qualitative findings will provide pilot data for future assessment of the InDI measures in other ethnoracial groups. Other future directions for this research include secondary use of the data (to be made publicly available) for intersectional analyses of relationships between discrimination and health among SGM persons of color, for whom large publicly available data sets are scarce.

## Appendix

Multimedia Appendix 1 Intersectional Discrimination Index test measures.

Multimedia Appendix 2 Peer-reviewer report from the Health Disparities and Equity Promotion Study Section, National Institutes of Health.

## Abbreviations

CAPTCHA: Completely Automated Public Turing test to tell Computers and Humans Apart
CFA: confirmatory factor analysis
InDI: Intersectional Discrimination Index
InDI-A: Intersectional Discrimination Index-anticipated
InDI-D: Intersectional Discrimination Index-day-to-day
InDI-M: Intersectional Discrimination Index-major
mCFA: multigroup confirmatory factor analysis
MIMIC: Multiple Indicator Multiple Cause
SGM: sexual and gender minority
SSP: social status and position
