# Supplementary material for: Advancing Intersectional Discrimination Measures for Health Disparities Research: Protocol for a Bilingual Mixed Methods Measurement Study
Source: JMIR Res Protoc. 2021 Aug 30;10(8):e30987. doi: 10.2196/30987 (PMC8438612; doi:10.2196/30987)
Supplement: Multimedia Appendix 1 [file resprot_v10i8e30987_app1.docx]

**SECTION 1 (Unattributed – All participants)**

These questions are about experiences related to **who you are**. This includes both how you describe yourself and how others might describe you. For example, your skin color, ancestry, nationality, religion, gender, sexuality, age, weight, disability or mental health issue, and income.

|  | Strongly  disagree | | Disagree | Neither agree nor disagree | | Agree | | | Strongly agree |
| --- | --- | --- | --- | --- | --- | --- | --- | --- | --- |
| 1. Because of who I am, a doctor or nurse, or other health care provider might treat me poorly. | |  | | |  | |  |  |  |
| 1. Because of who I am, I might have trouble finding or keeping a job. | |  | | |  | |  |  |  |
| 1. Because of who I am, I might have trouble getting an apartment or house. | |  | | |  | |  |  |  |
| 1. I worry about being treated unfairly by a teacher, supervisor, or employer. | |  | | |  | |  |  |  |
| 1. I may be denied a bank account, loan, or mortgage because of who   I am. | |  | | |  | |  |  |  |
| 1. I worry about being harassed or stopped by police or security. | |  | | |  | |  |  |  |
| 1. Because of who I am, people might try to attack me physically. | |  | | |  | |  |  |  |
| 1. I expect to be pointed at, called names, or harassed when in public. | |  | | |  | |  |  |  |
| 1. I fear that I will have a hard time finding friendship or romance because of who I am. | |  | | |  | |  |  |  |

**Because of who you are, have you…**

|  | Never | Yes, but not in the past year | Yes, once or twice in the past year | Yes, many times in the past year |
| --- | --- | --- | --- | --- |
| 1. Heard, saw, or read others joking or laughing about you (or people like you) |  |  |  |  |
| 1. Been treated as if you are unfriendly, unhelpful, or rude |  |  |  |  |
| 1. Been called names or heard/saw your identity used as an insult |  |  |  |  |
| 1. Been treated as if others are afraid of you |  |  |  |  |
| 1. Been stared or pointed at in public |  |  |  |  |
| 1. Been told that you should think, act, or look more like others |  |  |  |  |
| 1. Heard that you or people like you don’t belong |  |  |  |  |
| 1. Asked inappropriate, offensive, or overly personal questions |  |  |  |  |
| 1. Been treated as if you are less smart or capable than others |  |  |  |  |

As a reminder, we are interested in experiences related to **who you are**. This includes both how you describe yourself and how others might describe you. For example, your skin color, ancestry, nationality, religion, gender, sexuality, age, weight, disability or mental health issue, and income.

1. Because of who you are, has a health care provider ever refused you care?

Never

Once

More than once

🡪 *If once or more*: Has this happened to you in the past 12 months?  Yes  No

1. Because of who you are, have you ever been fired or dismissed from a job, or been turned down for a job that you interviewed for?

Never

Once

More than once

🡪 *If once or more*: Has this happened to you in the past 12 months?  Yes  No

1. Because of who you are, have you ever been evicted or denied housing?

Never

Once

More than once

🡪 *If once or more*: Has this happened to you in the past 12 months?  Yes  No

1. Because of who you are, have you ever been unreasonably stopped and questioned, searched, or arrested by police or security?

Never

Once

More than once

🡪 *If once or more*: Has this happened to you in the past 12 months?  Yes  No

1. Because of who you are, have you ever been unreasonably expelled or suspended from school?

Never

Once

More than once

🡪 *If once or more*: Has this happened to you in the past 12 months?  Yes  No

1. Because of who you are, have you ever been unable to open a bank account, cash a cheque, or get a loan?

Never

Once

More than once

🡪 *If once or more*: Has this happened to you in the past 12 months?  Yes  No

1. Because of who you are, have you ever had to move to another neighborhood, town, city, state, province, or country?

Never

Once

More than once

🡪 *If once or more*: Has this happened to you in the past 12 months?  Yes  No

1. Because of who you are, have you ever lost a close relationship (e.g., with a family member, friend, or partner)?

Never

Once

More than once

🡪 *If once or more*: Has this happened to you in the past 12 months?  Yes  No

1. Because of who you are, have you ever been repeatedly harassed at work or school, where you live, or when accessing services?

No

Yes—in one place

Yes—in more than one place

🡪 *If in one place or more*: Has this happened to you in the past 12 months?  Yes  No

1. Because of who you are, have you ever been threatened with a physical or sexual attack?

Never

Once

More than once

🡪 *If once or more*: Has this happened to you in the past 12 months?  Yes  No

1. Because of who you are, have you ever been physically attacked (e.g., spit on, had objects thrown at you, hit, punched, pushed or grabbed, beaten)?

Never

Once

More than once

🡪 *If once or more*: Has this happened to you in the past 12 months?  Yes  No

1. Because of who you are, have you ever been made to engage in sexual activity, or been touched in a sexual way, that you didn’t want?

Never

Once

More than once

🡪 *If once or more*: Has this happened to you in the past 12 months?  Yes  No

1. Because of who you are, have you ever had someone take, damage, or vandalize your property?

Never

Once

More than once

🡪 *If once or more*: Has this happened to you in the past 12 months?  Yes  No

**SECTION 2 (Overall attributed – Random half of participants)**

These questions are about experiences related to **who you are**. This includes both how you describe yourself and how others might describe you. For example, your skin color, ancestry, nationality, religion, gender, sexuality, age, weight, disability or mental health issue, and income.

|  | Strongly  disagree | | Disagree | Neither agree nor disagree | | Agree | | | Strongly agree |
| --- | --- | --- | --- | --- | --- | --- | --- | --- | --- |
| 1. Because of who I am, a doctor or nurse, or other health care provider might treat me poorly. | |  | | |  | |  |  |  |
| 1. Because of who I am, I might have trouble finding or keeping a job. | |  | | |  | |  |  |  |
| 1. Because of who I am, I might have trouble getting an apartment or house. | |  | | |  | |  |  |  |
| 1. I worry about being treated unfairly by a teacher, supervisor, or employer. | |  | | |  | |  |  |  |
| 1. I may be denied a bank account, loan, or mortgage because of who   I am. | |  | | |  | |  |  |  |
| 1. I worry about being harassed or stopped by police or security. | |  | | |  | |  |  |  |
| 1. Because of who I am, people might try to attack me physically. | |  | | |  | |  |  |  |
| 1. I expect to be pointed at, called names, or harassed when in public. | |  | | |  | |  |  |  |
| 1. I fear that I will have a hard time finding friendship or romance because of who I am. | |  | | |  | |  |  |  |

🡪 If answered “neither agree nor disagree”, ‘agree”, or “strongly agree” to one or more of items 1-9:

Thinking of the questions we just asked you, what parts of who you are might be a reason why others treat you unfairly?

- Your age
- Your gender
- Your transgender or gender non-conforming status
- Your sexual orientation (being gay, lesbian, bisexual, or straight)
- Your citizenship status or country of origin
- Your income
- Your level of education
- Your mental health issue or substance use
- Your physical disability
- Your race (skin color)
- Your ethnicity (cultural background or ancestry)
- Your religion
- Your language (e.g., accent, how you speak English, other language spoken)
- Your weight
- Another reason, please specify ____________

**Because of who you are, have you…**

|  | Never | Yes, but not in the past year | Yes, once or twice in the past year | Yes, many times in the past year |
| --- | --- | --- | --- | --- |
| 1. Heard, saw, or read others joking or laughing about you (or people like you) |  |  |  |  |
| 1. Been treated as if you are unfriendly, unhelpful, or rude |  |  |  |  |
| 1. Been called names or heard/saw your identity used as an insult |  |  |  |  |
| 1. Been treated as if others are afraid of you |  |  |  |  |
| 1. Been stared or pointed at in public |  |  |  |  |
| 1. Been told that you should think, act, or look more like others |  |  |  |  |
| 1. Heard that you or people like you don’t belong |  |  |  |  |
| 1. Asked inappropriate, offensive, or overly personal questions |  |  |  |  |
| 1. Been treated as if you are less smart or capable than others |  |  |  |  |

🡪 If “yes” to one or more of items 10-18:

Thinking of the questions we just asked you, why do you think others treated you unfairly? (check all that apply)

- Your age
- Your gender
- Your transgender or gender non-conforming status
- Your sexual orientation (being gay, lesbian, bisexual, or straight)
- Your citizenship status or country of origin
- Your income
- Your level of education
- Your mental health issue or substance use
- Your physical disability
- Your race (skin color)
- Your ethnicity (cultural background or ancestry)
- Your religion
- Your language (e.g., accent, how you speak English, other language spoken)
- Your weight
- Another reason, please specify ____________

As a reminder, we are interested in experiences related to **who you are**. This includes both how you describe yourself and how others might describe you. For example, your skin color, ancestry, nationality, religion, gender, sexuality, age, weight, disability or mental health issue, and income.

1. Because of who you are, has a health care provider ever refused you care?

Never

Once

More than once

🡪 *If once or more*: Has this happened to you in the past 12 months?  Yes  No

1. Because of who you are, have you ever been fired or dismissed from a job, or been turned down for a job that you interviewed for?

Never

Once

More than once

🡪 *If once or more*: Has this happened to you in the past 12 months?  Yes  No

1. Because of who you are, have you ever been evicted or denied housing?

Never

Once

More than once

🡪 *If once or more*: Has this happened to you in the past 12 months?  Yes  No

1. Because of who you are, have you ever been unreasonably stopped and questioned, searched, or arrested by police or security?

Never

Once

More than once

🡪 *If once or more*: Has this happened to you in the past 12 months?  Yes  No

1. Because of who you are, have you ever been unreasonably expelled or suspended from school?

Never

Once

More than once

🡪 *If once or more*: Has this happened to you in the past 12 months?  Yes  No

1. Because of who you are, have you ever been unable to open a bank account, cash a cheque, or get a loan?

Never

Once

More than once

🡪 *If once or more*: Has this happened to you in the past 12 months?  Yes  No

1. Because of who you are, have you ever had to move to another neighborhood, town, city, state, province, or country?

Never

Once

More than once

🡪 *If once or more*: Has this happened to you in the past 12 months?  Yes  No

1. Because of who you are, have you ever lost a close relationship (e.g., with a family member, friend, or partner)?

Never

Once

More than once

🡪 *If once or more*: Has this happened to you in the past 12 months?  Yes  No

1. Because of who you are, have you ever been repeatedly harassed at work or school, where you live, or when accessing services?

No

Yes—in one place

Yes—in more than one place

🡪 *If in one place or more*: Has this happened to you in the past 12 months?  Yes  No

1. Because of who you are, have you ever been threatened with a physical or sexual attack?

Never

Once

More than once

🡪 *If once or more*: Has this happened to you in the past 12 months?  Yes  No

1. Because of who you are, have you ever been physically attacked (e.g., spit on, had objects thrown at you, hit, punched, pushed or grabbed, beaten)?

Never

Once

More than once

🡪 *If once or more*: Has this happened to you in the past 12 months?  Yes  No

1. Because of who you are, have you ever been made to engage in sexual activity, or been touched in a sexual way, that you didn’t want?

Never

Once

More than once

🡪 *If once or more*: Has this happened to you in the past 12 months?  Yes  No

1. Because of who you are, have you ever had someone take, damage, or vandalize your property?

Never

Once

More than once

🡪 *If once or more*: Has this happened to you in the past 12 months?  Yes  No

🡪 If “yes” to one or more of items 19-31:

Thinking of the questions we just asked you, why do you think others treated you unfairly? (check all that apply)

- Your age
- Your gender
- Your transgender or gender non-conforming status
- Your sexual orientation (being gay, lesbian, bisexual, or straight)
- Your citizenship status or country of origin
- Your income
- Your level of education
- Your mental health issue or substance use
- Your physical disability
- Your race (skin color)
- Your ethnicity (cultural background or ancestry)
- Your religion
- Your language (e.g., accent, how you speak English, other language spoken)
- Your weight
- Another reason, please specify ____________

These questions are about experiences related to **who you are**. This includes both how you describe yourself and how others might describe you. For example, your skin color, ancestry, nationality, religion, gender, sexuality, age, weight, disability or mental health issue, and income.

|  | Strongly  disagree | | Disagree | Neither agree nor disagree | | Agree | | | Strongly agree |
| --- | --- | --- | --- | --- | --- | --- | --- | --- | --- |
| 1. Because of who I am, a doctor or nurse, or other health care provider might treat me poorly. | |  | | |  | |  |  |  |
| [*If “neither agree nor disagree”, ‘agree”, or “strongly agree”*]  Why might a doctor, nurse, or healthcare provider treat you poorly? (check all that apply)   - Your age - Your gender - Your transgender or gender non-conforming status - Your sexual orientation (being gay, lesbian, bisexual, or straight) - Your citizenship status or country of origin - Your income - Your level of education - Your mental health issue or substance use - Your physical disability - Your race (skin color) - Your ethnicity (cultural background or ancestry) - Your religion - Your language (e.g., accent, how you speak English, other language spoken) - Your weight - Another reason, please specify ____________ | | | | | | | | | |
| 1. Because of who I am, I might have trouble finding or keeping a job. | |  | | |  | |  |  |  |
| [*If “neither agree nor disagree”, ‘agree”, or “strongly agree”*]  Why might you have trouble finding or keeping a job? (check all that apply)   - Your age - Your gender - Your transgender or gender non-conforming status - Your sexual orientation (being gay, lesbian, bisexual, or straight) - Your citizenship status or country of origin - Your income - Your level of education - Your mental health issue or substance use - Your physical disability - Your race (skin color) - Your ethnicity (cultural background or ancestry) - Your religion - Your language (e.g., accent, how you speak English, other language spoken) - Your weight - Another reason, please specify ___________ | | | | | | | | | |
| 1. Because of who I am, I might have trouble getting an apartment or house. | |  | | |  | |  |  |  |
| [*If “neither agree nor disagree”, ‘agree”, or “strongly agree”*]  Why might you have trouble getting an apartment or house? (check all that apply)   - Your age - Your gender - Your transgender or gender non-conforming status - Your sexual orientation (being gay, lesbian, bisexual, or straight) - Your citizenship status or country of origin - Your income - Your level of education - Your mental health issue or substance use - Your physical disability - Your race (skin color) - Your ethnicity (cultural background or ancestry) - Your religion - Your language (e.g., accent, how you speak English, other language spoken) - Your weight - Another reason, please specify | | | | | | | | | |
| 1. I worry about being treated unfairly by a teacher, supervisor, or employer. | |  | | |  | |  |  |  |
| [*If “neither agree nor disagree”, ‘agree”, or “strongly agree”*]  Why might you be treated unfairly by a teacher, supervisor, or employer? (check all that apply)   - Your age - Your gender - Your transgender or gender non-conforming status - Your sexual orientation (being gay, lesbian, bisexual, or straight) - Your citizenship status or country of origin - Your income - Your level of education - Your mental health issue or substance use - Your physical disability - Your race (skin color) - Your ethnicity (cultural background or ancestry) - Your religion - Your language (e.g., accent, how you speak English, other language spoken) - Your weight - Another reason, please specify | | | | | | | | | |
| 1. I may be denied a bank account, loan, or mortgage because of who   I am. | |  | | |  | |  |  |  |
| [*If “neither agree nor disagree”, ‘agree”, or “strongly agree”*]  Why might you be denied a bank account, loan, or mortgage? (check all that apply)   - Your age - Your gender - Your transgender or gender non-conforming status - Your sexual orientation (being gay, lesbian, bisexual, or straight) - Your citizenship status or country of origin - Your income - Your level of education - Your mental health issue or substance use - Your physical disability - Your race (skin color) - Your ethnicity (cultural background or ancestry) - Your religion - Your language (e.g., accent, how you speak English, other language spoken) - Your weight - Another reason, please specify | | | | | | | | | |
| 1. I worry about being harassed or stopped by police or security. | |  | | |  | |  |  |  |
| [*If “neither agree nor disagree”, ‘agree”, or “strongly agree”*]  Why might you be harassed or stopped by police or security? (check all that apply)   - Your age - Your gender - Your transgender or gender non-conforming status - Your sexual orientation (being gay, lesbian, bisexual, or straight) - Your citizenship status or country of origin - Your income - Your level of education - Your mental health issue or substance use - Your physical disability - Your race (skin color) - Your ethnicity (cultural background or ancestry) - Your religion - Your language (e.g., accent, how you speak English, other language spoken) - Your weight - Another reason, please specify | | | | | | | | | |
| 1. Because of who I am, people might try to attack me physically. | |  | | |  | |  |  |  |
| [*If “neither agree nor disagree”, ‘agree”, or “strongly agree”*]  Why might you be attacked physically? (check all that apply)   - Your age - Your gender - Your transgender or gender non-conforming status - Your sexual orientation (being gay, lesbian, bisexual, or straight) - Your citizenship status or country of origin - Your income - Your level of education - Your mental health issue or substance use - Your physical disability - Your race (skin color) - Your ethnicity (cultural background or ancestry) - Your religion - Your language (e.g., accent, how you speak English, other language spoken) - Your weight - Another reason, please specify | | | | | | | | | |
| 1. I expect to be pointed at, called names, or harassed when in public. | |  | | |  | |  |  |  |
| [*If “neither agree nor disagree”, ‘agree”, or “strongly agree”*]  Why might you be pointed at, called names, or harassed when in public? (check all that apply)   - Your age - Your gender - Your transgender or gender non-conforming status - Your sexual orientation (being gay, lesbian, bisexual, or straight) - Your citizenship status or country of origin - Your income - Your level of education - Your mental health issue or substance use - Your physical disability - Your race (skin color) - Your ethnicity (cultural background or ancestry) - Your religion - Your language (e.g., accent, how you speak English, other language spoken) - Your weight - Another reason, please specify | | | | | | | | | |
| 1. I fear that I will have a hard time finding friendship or romance because of who I am. | |  | | |  | |  |  |  |
| [*If “neither agree nor disagree”, ‘agree”, or “strongly agree”*]  Why might you have a hard time finding friendship or romance? (check all that apply)   - Your age - Your gender - Your transgender or gender non-conforming status - Your sexual orientation (being gay, lesbian, bisexual, or straight) - Your citizenship status or country of origin - Your income - Your level of education - Your mental health issue or substance use - Your physical disability - Your race (skin color) - Your ethnicity (cultural background or ancestry) - Your religion - Your language (e.g., accent, how you speak English, other language spoken) - Your weight - Another reason, please specify | | | | | | | | | |

**Because of who you are, have you…**

|  | Never | Yes, but not in the past year | Yes, once or twice in the past year | Yes, many times in the past year |
| --- | --- | --- | --- | --- |
| 1. Heard, saw, or read others joking or laughing about you (or people like you) |  |  |  |  |
| [*If “Yes”*] Why do you think this happened? (check all that apply)   - Your age - Your gender - Your transgender or gender non-conforming status - Your sexual orientation (being gay, lesbian, bisexual, or straight) - Your citizenship status or country of origin - Your income - Your level of education - Your mental health issue or substance use - Your physical disability - Your race (skin color) - Your ethnicity (cultural background or ancestry) - Your religion - Your language (e.g., accent, how you speak English, other language spoken) - Your weight - Another reason, please specify ____________ | | | | |
| 1. Been treated as if you are unfriendly, unhelpful, or rude |  |  |  |  |
| [*If “Yes”*] Why do you think this happened? (check all that apply)   - Your age - Your gender - Your transgender or gender non-conforming status - Your sexual orientation (being gay, lesbian, bisexual, or straight) - Your citizenship status or country of origin - Your income - Your level of education - Your mental health issue or substance use - Your physical disability - Your race (skin color) - Your ethnicity (cultural background or ancestry) - Your religion - Your language (e.g., accent, how you speak English, other language spoken) - Your weight - Another reason, please specify ____________ | | | | |
| 1. Been called names or heard/saw your identity used as an insult |  |  |  |  |
| [*If “Yes”*] Why do you think this happened? (check all that apply)   - Your age - Your gender - Your transgender or gender non-conforming status - Your sexual orientation (being gay, lesbian, bisexual, or straight) - Your citizenship status or country of origin - Your income - Your level of education - Your mental health issue or substance use - Your physical disability - Your race (skin color) - Your ethnicity (cultural background or ancestry) - Your religion - Your language (e.g., accent, how you speak English, other language spoken) - Your weight - Another reason, please specify ____________ | | | | |
| 1. Been treated as if others are afraid of you |  |  |  |  |
| [*If “Yes”*] Why do you think this happened? (check all that apply)   - Your age - Your gender - Your transgender or gender non-conforming status - Your sexual orientation (being gay, lesbian, bisexual, or straight) - Your citizenship status or country of origin - Your income - Your level of education - Your mental health issue or substance use - Your physical disability - Your race (skin color) - Your ethnicity (cultural background or ancestry) - Your religion - Your language (e.g., accent, how you speak English, other language spoken) - Your weight - Another reason, please specify ____________ | | | | |
| 1. Been stared or pointed at in public |  |  |  |  |
| [*If “Yes”*] Why do you think this happened? (check all that apply)   - Your age - Your gender - Your transgender or gender non-conforming status - Your sexual orientation (being gay, lesbian, bisexual, or straight) - Your citizenship status or country of origin - Your income - Your level of education - Your mental health issue or substance use - Your physical disability - Your race (skin color) - Your ethnicity (cultural background or ancestry) - Your religion - Your language (e.g., accent, how you speak English, other language spoken) - Your weight - Another reason, please specify ____________ | | | | |
| 1. Been told that you should think, act, or look more like others |  |  |  |  |
| [*If “Yes”*] Why do you think this happened? (check all that apply)   - Your age - Your gender - Your transgender or gender non-conforming status - Your sexual orientation (being gay, lesbian, bisexual, or straight) - Your citizenship status or country of origin - Your income - Your level of education - Your mental health issue or substance use - Your physical disability - Your race (skin color) - Your ethnicity (cultural background or ancestry) - Your religion - Your language (e.g., accent, how you speak English, other language spoken) - Your weight - Another reason, please specify ____________ | | | | |
| 1. Heard that you or people like you don’t belong |  |  |  |  |
| [*If “Yes”*] Why do you think this happened? (check all that apply)   - Your age - Your gender - Your transgender or gender non-conforming status - Your sexual orientation (being gay, lesbian, bisexual, or straight) - Your citizenship status or country of origin - Your income - Your level of education - Your mental health issue or substance use - Your physical disability - Your race (skin color) - Your ethnicity (cultural background or ancestry) - Your religion - Your language (e.g., accent, how you speak English, other language spoken) - Your weight - Another reason, please specify ____________ | | | | |
| 1. Asked inappropriate, offensive, or overly personal questions |  |  |  |  |
| [*If “Yes”*] Why do you think this happened? (check all that apply)   - Your age - Your gender - Your transgender or gender non-conforming status - Your sexual orientation (being gay, lesbian, bisexual, or straight) - Your citizenship status or country of origin - Your income - Your level of education - Your mental health issue or substance use - Your physical disability - Your race (skin color) - Your ethnicity (cultural background or ancestry) - Your religion - Your language (e.g., accent, how you speak English, other language spoken) - Your weight - Another reason, please specify ____________ | | | | |
| 1. Been treated as if you are less smart or capable than others |  |  |  |  |
| [*If “Yes”*] Why do you think this happened? (check all that apply)   - Your age - Your gender - Your transgender or gender non-conforming status - Your sexual orientation (being gay, lesbian, bisexual, or straight) - Your citizenship status or country of origin - Your income - Your level of education - Your mental health issue or substance use - Your physical disability - Your race (skin color) - Your ethnicity (cultural background or ancestry) - Your religion - Your language (e.g., accent, how you speak English, other language spoken) - Your weight - Another reason, please specify ____________ | | | | |

As a reminder, we are interested in experiences related to **who you are**. This includes both how you describe yourself and how others might describe you. For example, your skin color, ancestry, nationality, religion, gender, sexuality, age, weight, disability or mental health issue, and income.

1. Because of who you are, has a health care provider ever refused you care?

Never

Once

More than once

🡪 *If once or more*: Has this happened to you in the past 12 months?  Yes  No

🡪 *If once or more*: Why do you think this happened? (check all that apply)

- - Your age
  - Your gender
  - Your transgender or gender non-conforming status
  - Your sexual orientation (being gay, lesbian, bisexual, or straight)
  - Your citizenship status or country of origin
  - Your income
  - Your level of education
  - Your mental health issue or substance use
  - Your physical disability
  - Your race (skin color)
  - Your ethnicity (cultural background or ancestry)
  - Your religion
  - Your language (e.g., accent, how you speak English, other language spoken)
  - Your weight
  - Another reason, please specify ____________

1. Because of who you are, have you ever been fired or dismissed from a job, or been turned down for a job that you interviewed for?

Never

Once

More than once

🡪 *If once or more*: Has this happened to you in the past 12 months?  Yes  No

🡪 *If once or more*: Why do you think this happened? (check all that apply)

- - Your age
  - Your gender
  - Your transgender or gender non-conforming status
  - Your sexual orientation (being gay, lesbian, bisexual, or straight)
  - Your citizenship status or country of origin
  - Your income
  - Your level of education
  - Your mental health issue or substance use
  - Your physical disability
  - Your race (skin color)
  - Your ethnicity (cultural background or ancestry)
  - Your religion
  - Your language (e.g., accent, how you speak English, other language spoken)
  - Your weight
  - Another reason, please specify ____________

1. Because of who you are, have you ever been evicted or denied housing?

Never

Once

More than once

🡪 *If once or more*: Has this happened to you in the past 12 months?  Yes  No

🡪 *If once or more*: Why do you think this happened? (check all that apply)

- - Your age
  - Your gender
  - Your transgender or gender non-conforming status
  - Your sexual orientation (being gay, lesbian, bisexual, or straight)
  - Your citizenship status or country of origin
  - Your income
  - Your level of education
  - Your mental health issue or substance use
  - Your physical disability
  - Your race (skin color)
  - Your ethnicity (cultural background or ancestry)
  - Your religion
  - Your language (e.g., accent, how you speak English, other language spoken)
  - Your weight
  - Another reason, please specify ____________

1. Because of who you are, have you ever been unreasonably stopped and questioned, searched, or arrested by police or security?

Never

Once

More than once

🡪 *If once or more*: Has this happened to you in the past 12 months?  Yes  No

🡪 *If once or more*: Why do you think this happened? (check all that apply)

- - Your age
  - Your gender
  - Your transgender or gender non-conforming status
  - Your sexual orientation (being gay, lesbian, bisexual, or straight)
  - Your citizenship status or country of origin
  - Your income
  - Your level of education
  - Your mental health issue or substance use
  - Your physical disability
  - Your race (skin color)
  - Your ethnicity (cultural background or ancestry)
  - Your religion
  - Your language (e.g., accent, how you speak English, other language spoken)
  - Your weight
  - Another reason, please specify ____________

1. Because of who you are, have you ever been unreasonably expelled or suspended from school?

Never

Once

More than once

🡪 *If once or more*: Has this happened to you in the past 12 months?  Yes  No

🡪 *If once or more*: Why do you think this happened? (check all that apply)

- - Your age
  - Your gender
  - Your transgender or gender non-conforming status
  - Your sexual orientation (being gay, lesbian, bisexual, or straight)
  - Your citizenship status or country of origin
  - Your income
  - Your level of education
  - Your mental health issue or substance use
  - Your physical disability
  - Your race (skin color)
  - Your ethnicity (cultural background or ancestry)
  - Your religion
  - Your language (e.g., accent, how you speak English, other language spoken)
  - Your weight
  - Another reason, please specify ____________

1. Because of who you are, have you ever been unable to open a bank account, cash a cheque, or get a loan?

Never

Once

More than once

🡪 *If once or more*: Has this happened to you in the past 12 months?  Yes  No

🡪 *If once or more*: Why do you think this happened? (check all that apply)

- - Your age
  - Your gender
  - Your transgender or gender non-conforming status
  - Your sexual orientation (being gay, lesbian, bisexual, or straight)
  - Your citizenship status or country of origin
  - Your income
  - Your level of education
  - Your mental health issue or substance use
  - Your physical disability
  - Your race (skin color)
  - Your ethnicity (cultural background or ancestry)
  - Your religion
  - Your language (e.g., accent, how you speak English, other language spoken)
  - Your weight
  - Another reason, please specify ____________

1. Because of who you are, have you ever had to move to another neighborhood, town, city, state, province, or country?

Never

Once

More than once

🡪 *If once or more*: Has this happened to you in the past 12 months?  Yes  No

🡪 *If once or more*: Why do you think this happened? (check all that apply)

- - Your age
  - Your gender
  - Your transgender or gender non-conforming status
  - Your sexual orientation (being gay, lesbian, bisexual, or straight)
  - Your citizenship status or country of origin
  - Your income
  - Your level of education
  - Your mental health issue or substance use
  - Your physical disability
  - Your race (skin color)
  - Your ethnicity (cultural background or ancestry)
  - Your religion
  - Your language (e.g., accent, how you speak English, other language spoken)
  - Your weight
  - Another reason, please specify ____________

1. Because of who you are, have you ever lost a close relationship (e.g., with a family member, friend, or partner)?

Never

Once

More than once

🡪 *If once or more*: Has this happened to you in the past 12 months?  Yes  No

🡪 *If once or more*: Why do you think this happened? (check all that apply)

- - Your age
  - Your gender
  - Your transgender or gender non-conforming status
  - Your sexual orientation (being gay, lesbian, bisexual, or straight)
  - Your citizenship status or country of origin
  - Your income
  - Your level of education
  - Your mental health issue or substance use
  - Your physical disability
  - Your race (skin color)
  - Your ethnicity (cultural background or ancestry)
  - Your religion
  - Your language (e.g., accent, how you speak English, other language spoken)
  - Your weight
  - Another reason, please specify ____________

1. Because of who you are, have you ever been repeatedly harassed at work or school, where you live, or when accessing services?

No

Yes—in one place

Yes—in more than one place

🡪 *If in one place or more*: Has this happened to you in the past 12 months?  Yes  No

🡪 *If once or more*: Why do you think this happened? (check all that apply)

- - Your age
  - Your gender
  - Your transgender or gender non-conforming status
  - Your sexual orientation (being gay, lesbian, bisexual, or straight)
  - Your citizenship status or country of origin
  - Your income
  - Your level of education
  - Your mental health issue or substance use
  - Your physical disability
  - Your race (skin color)
  - Your ethnicity (cultural background or ancestry)
  - Your religion
  - Your language (e.g., accent, how you speak English, other language spoken)
  - Your weight
  - Another reason, please specify ____________

1. Because of who you are, have you ever been threatened with a physical or sexual attack?

Never

Once

More than once

🡪 *If once or more*: Has this happened to you in the past 12 months?  Yes  No

🡪 *If once or more*: Why do you think this happened? (check all that apply)

- - Your age
  - Your gender
  - Your transgender or gender non-conforming status
  - Your sexual orientation (being gay, lesbian, bisexual, or straight)
  - Your citizenship status or country of origin
  - Your income
  - Your level of education
  - Your mental health issue or substance use
  - Your physical disability
  - Your race (skin color)
  - Your ethnicity (cultural background or ancestry)
  - Your religion
  - Your language (e.g., accent, how you speak English, other language spoken)
  - Your weight
  - Another reason, please specify ____________

1. Because of who you are, have you ever been physically attacked (e.g., spit on, had objects thrown at you, hit, punched, pushed or grabbed, beaten)?

Never

Once

More than once

🡪 *If once or more*: Has this happened to you in the past 12 months?  Yes  No

🡪 *If once or more*: Why do you think this happened? (check all that apply)

- - Your age
  - Your gender
  - Your transgender or gender non-conforming status
  - Your sexual orientation (being gay, lesbian, bisexual, or straight)
  - Your citizenship status or country of origin
  - Your income
  - Your level of education
  - Your mental health issue or substance use
  - Your physical disability
  - Your race (skin color)
  - Your ethnicity (cultural background or ancestry)
  - Your religion
  - Your language (e.g., accent, how you speak English, other language spoken)
  - Your weight
  - Another reason, please specify ____________

1. Because of who you are, have you ever been made to engage in sexual activity, or been touched in a sexual way, that you didn’t want?

Never

Once

More than once

🡪 *If once or more*: Has this happened to you in the past 12 months?  Yes  No

🡪 *If once or more*: Why do you think this happened? (check all that apply)

- - Your age
  - Your gender
  - Your transgender or gender non-conforming status
  - Your sexual orientation (being gay, lesbian, bisexual, or straight)
  - Your citizenship status or country of origin
  - Your income
  - Your level of education
  - Your mental health issue or substance use
  - Your physical disability
  - Your race (skin color)
  - Your ethnicity (cultural background or ancestry)
  - Your religion
  - Your language (e.g., accent, how you speak English, other language spoken)
  - Your weight
  - Another reason, please specify ____________

1. Because of who you are, have you ever had someone take, damage, or vandalize your property?

Never

Once

More than once

🡪 *If once or more*: Has this happened to you in the past 12 months?  Yes  No

🡪 *If once or more*: Why do you think this happened? (check all that apply)

- - Your age
  - Your gender
  - Your transgender or gender non-conforming status
  - Your sexual orientation (being gay, lesbian, bisexual, or straight)
  - Your citizenship status or country of origin
  - Your income
  - Your level of education
  - Your mental health issue or substance use
  - Your physical disability
  - Your race (skin color)
  - Your ethnicity (cultural background or ancestry)
  - Your religion
  - Your language (e.g., accent, how you speak English, other language spoken)
  - Your weight
  - Another reason, please specify ____________
