# Supplementary material for: Advancing Intersectional Discrimination Measures for Health Disparities Research: Protocol for a Bilingual Mixed Methods Measurement Study
Source: JMIR Res Protoc. 2021 Aug 30;10(8):e30987. doi: 10.2196/30987 (PMC8438612; doi:10.2196/30987)
Supplement: Multimedia Appendix 2 [file resprot_v10i8e30987_app2.pdf]

SUMMARY STATEMENT
(Privileged Communication)

PROGRAM CONTACT:
Jennifer Alvidrez
301-594-9567
alvidrezjl@mail.nih.gov

Release Date: 11/25/2020
Revised Date:

Application Number: 1 R21 MD016177-01

Principal Investigator

SCHEIM, AYDEN I

Applicant Organization: DREXEL UNIVERSITY

Review Group: HDEP
Health Disparities and Equity Promotion Study Section

Meeting Date: 10/28/2020
Council: JAN 2021
Requested Start: 04/01/2021
RFA/PA: PAR20-150
PCC: CPS02JA

Project Title: Advancing intersectional discrimination measures for health disparities research

SRG Action: Impact Score:20 Percentile:5 +
Next Steps: Visit https://grants.nih.gov/grants/next_steps.htm
Human Subjects: 30-Human subjects involved - Certified, no SRG concerns
Animal Subjects: 10-No live vertebrate animals involved for competing appl.
Gender: 1A-Both genders, scientifically acceptable
Minority: 1A-Minorities and non-minorities, scientifically acceptable
Age: 3A-No children included, scientifically acceptable

Project Year	Direct Costs Requested	Estimated Total Cost
1	175,000	273,899
2	100,000	156,513
TOTAL	275,000	430,412

ADMINISTRATIVE BUDGET NOTE: The budget shown is the requested budget and has not been adjusted to reflect any recommendations made by reviewers. If an award is planned, the costs will be calculated by Institute grants management staff based on the recommendations outlined below in the COMMITTEE BUDGET RECOMMENDATIONS section.

1R21MD016177-01 Scheim, Ayden

RESUME AND SUMMARY OF DISCUSSION: This application proposes to evaluate and test improvements in English and Spanish for the novel Intersectional Discrimination Index (InDI). Given the evidence about the impact of multiple forms of discrimination on health among racial/ethnic and sexual and gender minority populations, this is a highly significant area of inquiry. The panel noted a number of additional strengths including the rigorous examination of prior research to support the study's significance, strong investigative team, well described recruitment plan, and rigorous approach appropriately guided by a framework of intersectionality. The ability to test attributions for experiences of discrimination and the development of a Spanish version of the instrument are major strengths of the study. Minor weaknesses identified by reviewers include limited justification for the number of cognitive interviews and limitations in the sampling plan given the reliance on Facebook and social media. Some reviewers questioned the incremental value added of the study given the novel InDI recently developed by the investigative team. Others found the ability to test for comparative validity of the instrument and the Spanish language version as appropriate given noted gaps in the literature. Overall, the panel was enthusiastic about the study's high potential to advance intersectional health disparities research with validated measures of discrimination.

DESCRIPTION (provided by applicant): Guided by intersectionality frameworks, health disparities researchers have documented health disparities at the intersection of multiple axes of social status and position (SSP), particularly race/ethnicity, gender, and sexual orientation. To advance from identifying to intervening upon such intersectional health disparities, studies that examine underlying mechanisms are required. Much research demonstrates the negative health impacts of perceived discrimination within single health disparity populations. Quantitative approaches to assessing the role of discrimination in generating intersectional health disparities remain in their infancy, however. Members of our team recently introduced the Intersectional Discrimination Index (InDI) to address this gap. The InDI comprises three measures of enacted (day-to-day and major) and anticipated discrimination; these attribution-free measures ask about experiences of mistreatment "because of who you are." These measures show promise for intersectional health disparities research but require further validation across intersectional groups and languages. Additionally, the proposal to remove attributions is controversial and no direct comparison has been conducted. Therefore, this study aims to (1) cognitively and (2) psychometrically evaluate the Intersectional Discrimination Index (InDI) in English and Spanish and (3) determine whether attributions should be included. Study aims will draw on three original sequentially collected sources of data: (a) Qualitative cognitive interviews in English and Spanish (n=50) with a sample purposively recruited across intersecting SSP (gender, sexual orientation, race/ethnicity, socio-economic status, age, nativity); (b) a Spanish quantitative survey (n=500; 50% SGM); and (c) an English quantitative survey (n=3000), with quota sampling by race/ethnicity (Black, Latinx, White), SGM status, and gender. The study's key deliverable will be bilingual measures of anticipated, day-to-day, and major discrimination validated for multiple health disparity populations using rigorous qualitative, quantitative, and mixed methods. This expected outcome will support NIMHD priorities for Health Disparities Science by strengthening measurement of discrimination in population health research, thereby improving understanding of how it contributes to health disparities.

PUBLIC HEALTH RELEVANCE: The proposed study seeks to further develop instruments used to measure the impacts of multiple and intersectional discrimination on health and health disparities, in alignment with NIMHD's prioritization of research to improve measurement of racism and other forms of discrimination. The study will deliver English and Spanish-language measures of anticipated, day-to-day, and major forms of discrimination validated for multiple health disparity populations. These measures, in turn, will be used to advance knowledge about how discrimination impacts health

disparities across intersecting axes of social status and position including race/ethnicity, gender, and sexual orientation.

CRITIQUE 1

Significance: 2
Investigator(s): 2
Innovation: 2
Approach: 2
Environment: 1

Overall Impact: The proposed study is likely to have high impact. Many researchers recognize the relevance of intersectionality theory for health equity research, but methods for assessing intersectional forms of discrimination are not well developed. This well-designed study will test improvements to a newly published instrument that made a splash when it appeared in 2019, resulting in the first bilingual, cognitively tested intersectional discrimination measure.

1. Significance:
Strengths
- Relevance of intersectionality theory to health equity is clear, but existing measures and analytical approaches are underdeveloped.
- Multidimensional approach to measurement of discrimination—including anticipatory, everyday, and major discrimination—builds on best available evidence.
- Relevance of attributions for discrimination remains a contested area of research in need of attention.
- Developing measure for Spanish speakers is important because of known differences in response patterns to other measures of discrimination among language groups.

Weaknesses
- It would be helpful to have a stronger conceptual framing of how cultural differences may relate to interpretation of and response to discrimination measures.

2. Investigator(s):
Strengths
- This team developed the original InDI, so is eminently qualified for the work.
- The investigators invited the author of a commentary on their original publication of InDI to join the team, which is exemplary and likely to enhance impact.
- Strong track record of collaboration and publication on research tightly linked to the aims of the current study.
- Good expertise on transgender health, relevant to intersectional framework.

Weaknesses
- None noted.

3. Innovation:
Strengths
- This study will have a significant impact on innovating measurement of discrimination in health research.
- Surprisingly, few studies have combined cognitive interviewing and psychometric evaluation of discrimination measures.

- The study will advance our understanding of whether and how to measure attributions of discrimination.

Weaknesses

- None noted.

4. Approach:
Strengths

- Solid plan for cognitive interviews in terms of data collection and analysis.
- Feasible recruiting plan, based on prior research.
- Good use of item response theory techniques.

Weaknesses

- Inadequate justification provided for sample size in cognitive interviews. Each subgroup (English and Spanish speakers) will encompass significant heterogeneity for gender, sexual orientation, race/ethnicity, socio-economic status, age, and nativity. Even if each of these criteria were treated a binary (an oversimplification), there would be 64 possible combinations. The consequence is that most intersections of categorical social status and position indicators will not be represented. The researchers appeal to the criterion of saturation for determining sample size, but given that more than half of intersectional identities will be represented, it is unlikely they will be able to determine whether they have reached saturation of not (see here the emerging literature critiquing and trying to operationalize saturation). This is the major score-driving weakness in my evaluation.

5. Environment:
Strengths

- Good support for research.
- Adequate facilities.

Weaknesses

- None noted.

Study Timeline:
Strengths

- None noted.

Weaknesses

- None noted.

Protections for Human Subjects:
Acceptable Risks and/or Adequate Protections
Data and Safety Monitoring Plan (Applicable for Clinical Trials Only):
Not Applicable (No Clinical Trials)

Inclusion Plans:

- Sex/Gender: Distribution justified scientifically
- Race/Ethnicity: Distribution justified scientifically
- For NIH-Defined Phase III trials, Plans for valid design and analysis: Not applicable
- Inclusion/Exclusion Based on Age: Distribution justified scientifically
- Acceptable.

Vertebrate Animals:
Not Applicable (No Vertebrate Animals)

Biohazards:
Not Applicable (No Biohazards)

Resource Sharing Plans:
Not Applicable (No Relevant Resources)

Budget and Period of Support:
Recommend as Requested

CRITIQUE 2

Significance: 2
Investigator(s): 1
Innovation: 2
Approach: 2
Environment: 2

Overall Impact: This proposal seeks to generate the first bilingual, cognitively evaluated intersectional discrimination measure and generate psychometric properties across intersectional subgroups. The proposal will test the already developed Intersectional Discrimination Index (InDI). The study's key deliverable will be a bilingual measure of anticipated, day-to-day, and major discrimination validated for multiple health disparity populations using a systematic and cumulative approach using qualitative, quantitative, and mixed methods. The weaknesses of the application are in the area of justification for the qualitative sample size in phase 1 and the need for more information on how qualitative data will be analyzed and synthesized with the data collected in phase 3, and the sample to be collected. The strengths of the project outweigh the weaknesses and the impact is positive for the outcomes of the proposed psychometric evaluation of the IDI.

1. Significance:
Strengths
- Intersectionality of race/ethnicity, gender, and sexual orientation is related to significant health disparities.
- Perceived discrimination is a preventable contributor to poor health outcomes.
- There is an inherent weakness in using within-in group analyses to demonstrate between group differences.
- Measurement of intercategorical intersectionality is nascent.

Weaknesses
- The measurement tool has already been developed and cognitively tested in previously funded efforts and is being used in other NIH funded projects.

2. Investigator(s):
Strengths
- The investigators have experience in the methods and content area to be addressed in this proposal.

Weaknesses
- None noted.

3. Innovation:

Strengths
- A measurement tool that is a bilingual measure of discrimination (day to day and major) that has been validated using a mixed methods approach.
- Investigation of attribution of discrimination is a significant innovation to the measurement.

Weaknesses
- None noted.

4. Approach:
Strengths
- The incorporation of cognitive interviews as a means to evaluate the IDI constructs.
- The investigators have developed the Intersectional Discrimination Index in a previous study.
- The utilization of multiple methods to refine the IDI.
- Psychometric evaluation of the IDI in Spanish and English with a social media generated sample, thereby enhancing external validity of the evaluation.
- Systematic psychometric evaluation of the IDI with intersecting axes of social status and position.

Weaknesses
- It is unclear what is new to this proposal about portions of Aim 2 as it is presented being reliant on the existing data and analyses from previously funded efforts.
- Relative lack of justification of 50 cognitive interviews in Phase 1.
- Need for more information on how qualitative data will be analyzed and synthesized with the data collected in phase 3.
- Sample recruitment carries a significant selection bias.

5. Environment:
Strengths
- The environment is supportive of the project and will serve to ensure successful completion.

Weaknesses
- None noted.

Study Timeline:
Strengths
- The timeline is feasible and realistic for the completion of the study aims

Weaknesses
- None noted.

Protections for Human Subjects:
Acceptable Risks and/or Adequate Protections
- The methods have been successfully used by the investigators in previous efforts. Aim 2 uses existing data.

Data and Safety Monitoring Plan (Applicable for Clinical Trials Only):
Not Applicable (No Clinical Trials)

Inclusion Plans:
- Sex/Gender: Distribution justified scientifically
- Race/Ethnicity: Distribution justified scientifically
- For NIH-Defined Phase III trials, Plans for valid design and analysis: Not applicable
- Inclusion/Exclusion Based on Age: Distribution justified scientifically

- Based on the proposed aims and activities the inclusion/exclusion and distribution are justified.

Vertebrate Animals:
Not Applicable (No Vertebrate Animals)

Biohazards:
Not Applicable (No Biohazards)

Resource Sharing Plans:
Not Applicable (No Relevant Resources)

Budget and Period of Support:
Recommend as Requested

CRITIQUE 3

Significance: 2
Investigator(s): 1
Innovation: 2
Approach: 2
Environment: 1

Overall Impact: There is a need for a more robust, efficient, and valid instrument to evaluate intersectional discrimination and its impacts on health. The investigative team has developed and tested the InDI instrument for 'face' validity. The team is transdisciplinary, experienced, and have demonstrated their ability to complete this type of research. The proposed methods for refinement (cognitive interviews), adapting to Spanish, and conducting a intersectional comparative validation study to the approach currently and commonly used in this field (attributed discrimination) is well-developed and rigorous. The team has excellent experience in conducting such studies with recruitment from the Web and other areas. Sex as a biologic variable is addressed. This is a strong proposal which could move measurement and attribution in the field of intersectional discrimination forward.

1. Significance:
Strengths
- Perceived, self-reported discrimination is associated with worse mental and physical health outcomes.
- Current instruments to assess discrimination are grounded in the type of discrimination being examined such as racial/ethnic, sexual gender minority, etc.. However, this is problematic when trying to assess intersectionality as individuals are not monolithic and may be experiencing perceived discrimination based on multiple individual characteristics. Currently, this requires multiple instruments, one for each type of discrimination and each with its own battery of questions.
- A new approach to address this was developed by the investigative team whereby overall perceived discrimination is examined in the Intersectional Discrimination Index. Types of discrimination are based on anticipated, day-to-day (i.e. acute) and major. These constructs are then crossed with demographic and characteristic information to examine social status and position discrimination.

- Prior work suggest this scale has excellent utility but it needs greater validation and needs to be designed for Spanish-speaking communities.

Weaknesses

- None noted.

2. Investigator(s):

Strengths

- Strong transdisciplinary investigative team with prior collaborative experience in developing, implementing, and analyzing the InDI. Team members have expertise in psychometrics, methods to evaluate intersectionality, Latin culture and Spanish intersectionality, cardiovascular health, mediation decomposition methods, and cognitive and psychometric evaluation.

Weaknesses

- None noted.

3. Innovation:

Strengths

- Development of a 'universal' discrimination instruments, adapting it to Spanish, and validating it against existing methods to evaluate intersectional discrimination is important and novel.

Weaknesses

- None noted.

4. Approach:

Strengths

- The investigators are building from strong prior research in developing the InDI.
- Recruitment methodology is appropriate for this type of large survey studies and the investigators have a track record with this approach but … see below. Incentives are appropriate.
- The proposed sample with all the stratifications is ambitious for 2 years but appears doable based on prior experience.
- Cognitive interview approach as part of refining existing InDI and for adapting to Spanish is good.
- Survey randomization and implementation design is good. Stratifying type of survey (unattributed, item-level attributed, and overall attributed) within only English-speaking participants is reasonable at this time of the instrument development. Quota sampling is also good to ensure balance in racial and ethnic minority by sexual gender minority categories.
- Analysis plans for Aims 1, 2, and 3 are appropriate. The mixed methods analysis of Aim 3 is well described.
- Sample size estimates are good at approximately 200 participants per intersectional category.

Weaknesses

- (Minor/Moderate) It would be helpful to provide some description of potential biases within the participants based on the recruitment methods.
- (Moderate) How do you determine 'meaningful' comparison between attribution instruments and within groups.

5. Environment:

Strengths

- While the team is from across multiple institutions they have good experience working together and support from their institutions.

Weaknesses

- None noted.

Study Timeline:
Strengths
- None noted.

Weaknesses
- None noted.

Protections for Human Subjects:
Acceptable Risks and/or Adequate Protections
Data and Safety Monitoring Plan (Applicable for Clinical Trials Only):
Acceptable

Inclusion Plans:
- Sex/Gender: Distribution justified scientifically
- Race/Ethnicity: Distribution justified scientifically
- For NIH-Defined Phase III trials, Plans for valid design and analysis: Not applicable
- Inclusion/Exclusion Based on Age: Distribution justified scientifically

Vertebrate Animals:
Not Applicable (No Vertebrate Animals)

Biohazards:
Not Applicable (No Biohazards)

Resource Sharing Plans:
Acceptable

Budget and Period of Support:
Recommend as Requested

THE FOLLOWING SECTIONS WERE PREPARED BY THE SCIENTIFIC REVIEW OFFICER TO SUMMARIZE THE OUTCOME OF DISCUSSIONS OF THE REVIEW COMMITTEE, OR REVIEWERS' WRITTEN CRITIQUES, ON THE FOLLOWING ISSUES:

PROTECTION OF HUMAN SUBJECTS: ACCEPTABLE

INCLUSION OF WOMEN PLAN: ACCEPTABLE

INCLUSION OF MINORITIES PLAN: ACCEPTABLE

INCLUSION ACROSS THE LIFESPAN: ACCEPTABLE

COMMITTEE BUDGET RECOMMENDATIONS: The budget was recommended as requested.

Footnotes for 1 R21 MD016177-01; PI Name: Scheim, Ayden I

+ Derived from the range of percentile values calculated for the study section that reviewed this application.

NIH has modified its policy regarding the receipt of resubmissions (amended applications).See Guide Notice NOT-OD-18-197 at https://grants.nih.gov/grants/guide/notice-files/NOT-OD-18-197.html. The impact/priority score is calculated after discussion of an application by averaging the overall scores (1-9) given by all voting reviewers on the committee and multiplying by 10. The criterion scores are submitted prior to the meeting by the individual reviewers assigned to an application, and are not discussed specifically at the review meeting or calculated into the overall impact score. Some applications also receive a percentile ranking. For details on the review process, see http://grants.nih.gov/grants/peer_review_process.htm#scoring.

MEETING ROSTER

Health Disparities and Equity Promotion Study Section
Healthcare Delivery and Methodologies Integrated Review Group
CENTER FOR SCIENTIFIC REVIEW
HDEP
10/28/2020 - 10/30/2020

Notice of NIH Policy to All Applicants: Meeting rosters are provided for information purposes only. Applicant investigators and institutional officials must not communicate directly with study section members about an application before or after the review. Failure to observe this policy will create a serious breach of integrity in the peer review process, and may lead to actions outlined in NOT-OD-14-073 at https://grants.nih.gov/grants/guide/notice-files/NOT-OD-14-073.html and NOT-OD-15-106 at https://grants.nih.gov/grants/guide/notice-files/NOT-OD-15-106.html, including removal of the application from immediate review.

CHAIRPERSON(S)

ARRIOLA, KIMBERLY RUTH JACOB, PHD, MPH
PROFESSOR
DEPARTMENT OF BEHAVIORAL, SOCIAL, AND HEALTH EDUCATION SCIENCES
ROLLINS SCHOOL OF PUBLIC HEALTH OF EMORY UNIVERSITY
ATLANTA, GA 30322

MEMBERS

ALLEN, HEIDI LYNN, PHD
ASSOCIATE PROFESSOR
SCHOOL OF SOCIAL WORK
COLUMBIA UNIVERSITY
NEW YORK, NY 10027

ARCOLEO, KIMBERLY JOAN, PHD, MPH
RESEARCH PROFESSOR OF NURSING
UNIVERSITY OF RHODE ISLAND COLLEGE OF NURSING
PROVIDENCE, RI 02903

BOATRIGHT, DOWIN, MD *
ASSISTANT PROFESSOR
DEPARTMENT OF EMERGENCY MEDICINE
YALE SCHOOL OF MEDICINE
NEW HAVEN, CT 06519

BROWN, ARLEEN F, MD, PHD
PROFESSOR
DEPARTMENT OF MEDICINE
UNIVERSITY OF CALIFORNIA, LOS ANGELES
LOS ANGELES, CA 90024

CUNNINGHAM, PHILLIPPE BELTON, PHD
PROFESSOR
DEPARTMENT OF PSYCHIATRY AND BEHAVIORAL SCIENCE
CENTER FOR GLOBAL AND COMMUNITY HEALTH
FAMILY SERVICES RESEARCH CENTER
MEDICAL UNIVERSITY OF SOUTH CAROLINA
CHARLESTON, SC 29401

DESAI, JAY R, PHD
RESEARCH INVESTIGATOR
HEALTH PARTNERS INSTITUTE
BLOOMINGTON, MN 55425

DOOLEY, WILLIAM CHESNUT, MD, BS *
PROFESSOR
DEPARTMENT OF SURGERY
UNIVERSITY OF OKLAHOMA HEALTH SCIENCE CENTER
OKLAHOMA CITY, OK 73104

EVANS CUELLAR, ALISON, PHD, BA, MBA *
PROFESSOR
DEPARTMENT OF HEALTH ADMINISTRATION AND POLICY
COLLEGE OF HEALTH AND HUMAN SERVICES
GEORGE MASON UNIVERSITY
FAIRFAX, VA 22030

GEISSLER, KIMBERLEY LYNN H, PHD *
ASSISTANT PROFESSOR
DEPARTMENT OF HEALTH PROMOTION AND POLICY
SCHOOL OF PUBLIC HEALTH AND HEALTH SCIENCES
UNIVERSITY OF MASSACHUSETTS AMHERST
AMHERST, MA 01003

GIURGESCU, CARMEN, PHD, FAAN, RN
PROFESSOR
COLLEGE OF NURSING
UNIVERSITY OF CENTRAL FLORIDA
ORLANDO, FL 32186

GOLDBACH, JEREMY THOMAS, PHD
ASSOCIATE PROFESSOR
SCHOOL OF SOCIAL WORK
UNIVERSITY OF SOUTHERN CALIFORNIA
LOS ANGELES, CA 90089

GONZALEZ-GUARDA, ROSA MARIA, MPH, MSN, PHD
ASSOCIATE PROFESSOR
DOROTHY L POWELL TERM CHAIR OF NURSING
DUKE UNIVERSITY SCHOOL OF NURSING
DUKE UNIVERSITY
DURHAM, NC 27710

GOODNEY, PHILIP P, MD, MS *
PROFESSOR AND DIRECTOR OF VA OUTCOMES GROUP
DEPARTMENT OF SURGERY
SECTION OF VASCULAR SURGERY
DARTMOUTH HITCHCOCK MEDICAL CENTER
LEBANON, NH 03750

GRAVLEE, CLARENCE C, PHD
ASSOCIATE PROFESSOR
DEPARTMENT OF ANTHROPOLOGY
UNIVERSITY OF FLORIDA
GAINESVILLE, FL 32611

GUEVARA, JAMES P, MD, MPH
PROFESSOR
DEPARTMENT OF PEDIATRICS
PERELMAN SCHOOL OF MEDICINE
UNIVERSITY OF PENNSYLVANIA
PHILADELPHIA, PA 19104

JUDD, SUZANNE E, MPH, PHD
PROFESSOR
DEPARTMENT OF BIOSTATISTICS
SCHOOL OF PUBLIC HEALTH
UNIVERSITY OF ALABAMA AT BIRMINGHAM
BIRMINGHAM, AL 35205

KARNIK, NIRANJAN, MD, PHD
PROFESSOR AND ASSOCIATE DEAN
DEPARTMENT OF PSYCHIATRY AND BEHAVIORAL
SCIENCES
RUSH UNIVERSITY MEDICAL CENTER
CHICAGO, IL 60612

KO, LINDA K, PHD
ASSOCIATE PROFESSOR
DIVISION OF PUBLIC HEALTH SCIENCES
FRED HUTCHINSON CANCER RESEARCH CENTER
UNIVERSITY OF WASHINGTON
SEATTLE, WA 98195

KONIARIS, LEONIDAS G., MD *
PROFESSOR
DEPARTMENT OF SURGERY
INDIANA UNIVERSITY
INDIANAPOLIS, IN 46202

LIEU, TRACY A, MPH, MD *
DIRECTOR
DIVISION OF RESEARCH
KAISER PERMANENTE NORTHERN CALIFORNIA
OAKLAND, CA 94612

MARTIN, MICHELLE Y, PHD *
PROFESSOR
DEPARTMENT OF PREVENTIVE MEDICINE
CENTER FOR INNOVATION IN HEALTH EQUITY RESEARCH
COLLEGE OF MEDICINE
UNIVERSITY OF TENNESSEE HEALTH SCIENCE CENTER
MEMPHIS, TN 38163

MCDONNELL, KAREN ANN, BA, PHD *
ASSOCIATE PROFESSOR AND VICE CHAIR
DEPARTMENT OF PREVENTION AND COMMUNITY HEALTH
SCHOOL OF PUBLIC HEALTH
GEORGE WASHINGTON UNIVERSITY
WASHINGTON, DC 20037

MUDD, GIA, PHD, BSN, MSN, MPH *
ASSOCIATE PROFESSOR
DIRECTOR OF THE COMMUNITY ENGAGEMENT
AND RESEARCH CORE
CENTER FOR CLINICAL AND TRANSLATIONAL SCIENCE
UNIVERSITY OF KENTUCKY
LEXINGTON, KY 40536

NAAVAAL, SHILLPA, DDS, MS, MPH *
ASSISTANT PROFESSOR
SCHOOL OF DENTISTRY
VIRGINIA COMMONWEALTH UNIVERSITY
RICHMOND, VA 23298

NORMAND, SHARON-LISE TERESA, PHD, MS, BS *
PROFESSOR OF HEALTH CARE POLICY AND BIOSTATISTICS
DEPARTMENT OF HEALTH CARE POLICY
HARVARD MEDICAL SCHOOL
BOSTON, MA 02115

OLUWOYE, OLADUNNI, PHD *
ASSISTANT PROFESSOR
ELSON S. FLOYD COLLEGE OF MEDICINE
WASHINGTON STATE UNIVERSITY
SPOKANE, WA 99202

OWUSU, CYNTHIA, BS, MD, MS *
ASSOCIATE PROFESSOR OF MEDICINE
DIVISION OF HEMATOLOGY ONCOLOGY
CASE WESTERN RESERVE UNIVERSITY SCHOOL OF
MEDICINE
CLEVELAND, OH 44139

POLIVKA, BARBARA J, MSN, PHD, FAAN
ASSOCIATE DEAN FOR RESEARCH AND PROFESSOR
SCHOOL OF NURSING
UNIVERSITY OF KANSAS
KANSAS CITY, KS 66160

QUINN, GWENDOLYN P, PHD
ENDOWED PROFESSOR
DEPARTMENTS OF OBSTETRICS AND GYNECOLOGY
AND POPULATION HEALTH
SCHOOL OF MEDICINE
NEW YORK UNIVERSITY
NEW YORK, NY 10016

RASMUS, STACY M, PHD
DIRECTOR AND RESEARCH ASSOCIATE PROFESSOR
INSTITUTE OF ARCTIC BIOLOGY
CENTER FOR ALASKA NATIVE HEALTH RESEARCH
UNIVERSITY OF ALASKA FAIRBANKS
FAIRBANKS, AK 99775

REIS, JANET M, BA, PHD *
PROFESSOR
OFFICE OF RESEARCH
COLLEGE OF HEALTH SCIENCES
BOISE STATE UNIVERSITY
BOISE, ID 83712

ROSENZWEIG, MARGARET Q, PHD
DISTINGUISHED SERVICE PROFESSOR
DEPARTMENT OF ACUTE/TERTIARY CARE
SCHOOL OF NURSING
UNIVERSITY OF PITTSBURGH
PITTSBURGH, PA 15261

SHUMWAY, MARTHA, PHD
PROFESSOR
DEPARTMENT OF PSYCHIATRY AND BEHAVIORAL
SCIENCES
SCHOOL OF MEDICINE
UNIVERSITY OF CALIFORNIA SAN FRANCISCO
SAN FRANCISCO, CA 94143

SMITH, NATHAN GRANT, BA, MS, PHD *
ASSOCIATE PROFESSOR
CHAIR, DEPARTMENT OF PSYCHOLOGICAL, HEALTH,
AND LEARNING SCIENCES
UNIVERSITY OF HOUSTON
HOUSTON, TX 77204

SOULAKOVA, JULIA, BS, MA, PHD *
ASSOCIATE PROFESSOR
DEPARTMENT OF POPULATION HEALTH SCIENCES
COLLEGE OF MEDICINE
UNIVERSITY OF CENTRAL FLORIDA
ORLANDO, FL 32827

TANJASIRI, SORA P, DRPH, MPH
PROFESSOR
DEPARTMENT OF EPIDEMOLOGY
UNIVERSITY OF CALIFORNIA, IRVINE
IRVINE, CA 92697

THORPE, ROLAND J JR, PHD
PROFESSOR
DEPARTMENT OF HEALTH, BEHAVIOR, AND SOCIETY
JOHNS HOPKINS BLOOMBERG SCHOOL OF PUBLIC HEALTH
BALTIMORE, MD 21205

TROGDON, JUSTIN, PHD *
PROFESSOR
DEPARTMENT OF HEALTH POLICY AND MANAGEMENT
GILLINGS SCHOOL OF GLOBAL PUBLIC HEALTH
UNIVERSITY OF NORTH CAROLINA AT CHAPEL HILL
CHAPEL HILL, NC 27599

WENDEL, MONICA L, DRPH, MA *
PROFESSOR AND CHAIR OF HEALTH PROMOTION AND
BEHAIVORAL SCIENCES
ASSOCIATE DEAN FOR PUBLIC HEALTH PRACTICE
SCHOOL OF PUBLIC HEALTH AND INFORMATION SCIENCES
UNIVERSITY OF LOUISVILLE
LOUISVILLE, KY 40202

WILLIAMS, MICHAEL D, BA, MD *
ASSOCIATE PROFESSOR OF SURGERY AND DIRECTOR
DEPARTMENT OF SURGERY
EMERGENCY GENERAL SURGERY
UNIVERSITY OF VIRGINIA SCHOOL OF MEDICINE
CHARLOTTESVILLE, VA 22908

MAIL REVIEWER(S)

WILLIS, ALLIRIC I, MD
ASSOCIATE PROFESSOR OF SURGERY
DEPARTMENT OF SURGICAL ONCOLOGY
SIDNEY KIMMEL MEDICAL COLLEGE
THOMAS JEFFERSON UNIVERSITY
PHILADELPHIA, PA 19107

SCIENTIFIC REVIEW OFFICER

BELLINGER, JESSICA, PHD
SCIENTIFIC REVIEW OFFICER
CENTER FOR SCIENTIFIC OF REVIEW
NATIONAL INSTITUTES OF HEALTH
BETHESDA, MD 20892

EXTRAMURAL SUPPORT ASSISTANT

FREID, NICOLE TIFFANY
EXTRAMURAL SUPPORT ASSISTANT
CENTER FOR SCIENTIFIC REVIEW
NATIONAL INSTITUTES OF HEALTH
BETHESDA, MD 20892

* Temporary Member. For grant applications, temporary members may participate in the entire meeting or may review only selected applications as needed.

Consultants are required to absent themselves from the room during the review of any application if their presence would constitute or appear to constitute a conflict of interest.
